# Bioactive Protein Profile and Compositional Evolution of Donkey Milk Across Lactation Reflecting Its Nutritional and Functional Food Value

**DOI:** 10.3390/foods14244284

**Published:** 2025-12-12

**Authors:** Ana-Maria Plotuna, Ionela Hotea, Alexandra Ban-Cucerzan, Kalman Imre, Viorel Herman, Ileana Nichita, Ionela Popa, Emil Tîrziu

**Affiliations:** Faculty of Veterinary Medicine, University of Life Sciences “King Mihai I” from Timisoara, Calea Aradului, No. 119, 300645 Timisoara, Romania; anamaria.plotuna@usvt.ro (A.-M.P.); alexandracucerzan@usvt.ro (A.B.-C.); kalmanimre@usvt.ro (K.I.); viorelherman@usvt.ro (V.H.); ileananichita@usvt.ro (I.N.); ionela.popa@usvt.ro (I.P.); emiltirziu@usvt.ro (E.T.)

**Keywords:** donkey milk, lactation stage, bioactive proteins, nutritional quality, ELISA, functional food, milk composition

## Abstract

Donkey milk is increasingly recognized as a functional food due to its unique nutritional profile and richness in bioactive compounds. This longitudinal observational study investigated changes in both chemical composition (total solids, protein, fat, lactose, and ash) and immune-active proteins (lactoferrin, α-lactalbumin, β-lactoglobulin, and lysozyme) across lactation. A total of 153 donkey milk samples were collected from five farms from very early (1–3 days in milk) to late lactation (30–210 days in milk). Chemical composition was determined using mid-infrared spectroscopy, while the concentrations of the immune-active proteins were determined by ELISA Quantitative Sandwich. Chemical analysis showed high values in colostrum, including total solids (10.13%), protein (3.1%), and ash (0.73%), which declined progressively during lactation to 8.45%, 1.14%, and 0.64%, respectively. Fat varied modestly, between 0.55 and 0.25%, while lactose remained stable at 5.75–6.41%. In parallel, bioactive proteins measured between 31 and 210 days exhibited distinct trajectories. Lactoferrin increased from 0.07 to 0.14 mg/mL, α-lactalbumin peaked mid-lactation at 2.58 mg/mL (compared with 1.91 mg/mL early and 2.25 mg/mL late), β-lactoglobulin declined from 0.84 to 0.55 mg/mL, and lysozyme decreased from 0.95 mg/mL early to 0.64 mg/mL late. Across lactation, we observed dilution of total solids and protein, relatively stable lactose and fat, and distinct trajectories of lactoferrin, α-lactalbumin, β-lactoglobulin, and lysozyme, indicating that donkey milk modulates rather than loses its protective protein profile. These results refine reference values for donkey milk and support its nutraceutical relevance for human nutrition and health.

## 1. Introduction

Global interest in functional dairy is rising as clinicians and consumers seek foods that can complement the prevention or management of chronic conditions. Donkey milk (DM) has emerged as a promising candidate, not only in the case of cow’s milk protein allergy (CMPA) but also for a wider set of nutraceutical effects grounded in its distinctive macro- and micronutrient profile and dense array of bioactive components [1,2]. Contemporary reviews now frame DM as a functional food with potential benefits extending to immune support, antimicrobial defense, gut and metabolic health, and cardiometabolic risk modulation [3,4,5,6].

From a compositional perspective, DM is low in fat and total protein (roughly 0.3–1.8 g fat and 1.4–1.8 g protein per 100 mL), relatively high in lactose (6–7 g/100 mL), and characterized by a whey-dominant protein profile (≈55–65% of total protein) closer to human milk than to bovine milk [7]. It provides essential vitamins (B-complex, D, and E) and trace minerals (selenium, zinc) relevant to immune function and redox balance; for example, thiamine and niacin reach about 0.66 and 18.8 µM, respectively, vitamin D around 2.2 µg/100 mL, and selenium about 10–11 µg/100 g, while zinc is in the order of 2–3 mg/L [4,8,9]. Its fatty acid pattern is comparatively rich in unsaturated fatty acids (≈55% of total fatty acids, including ≈25–30% PUFA and an n-6:n-3 ratio around 2:1) and yields favorable atherogenic (≈1.1) and thrombogenic (≈0.7) indices, features often linked to cardiometabolic health [10,11,12,13].

The bioactive fraction of DM is rich in α-lactalbumin (ALA ≈ 1.8 g/L), β-lactoglobulin (BLG ≈ 3.7 g/L), lactoferrin (LF ≈ 0.08 g/L), and lysozyme (LZM ≈ 1.0 g/L), with the last one occurring at high concentrations for a domestic dairy species, alongside immunoglobulins and diverse peptides [3,5,14,15,16]. Collectively, these proteins contribute antimicrobial, anti-inflammatory, immunomodulatory, and gut-supporting activities [16,17,18,19,20,21,22], and gentle pasteurization (65 °C for 30 min) appears to preserve much of LZM’s activity [15]. Importantly, previous analytical studies indicate that their concentrations are dynamic rather than static, with higher levels typically observed in early lactation and progressive declines toward mid and late stages, potentially modulating the milk’s antimicrobial and immunomodulatory capacity over time [19,23,24]. This variability is seldom considered when assessing the nutraceutical potential of donkey milk, creating a gap in practical validation.

The present work quantifies the chemical composition of donkey milk and four sentinel bioactive compounds (ALA, LF, BLG, and LZM) across defined lactation stages, situates these values within published ranges, and discusses their implications for human nutrition and health. The novelty of this study lies in its integrated and comprehensive approach to characterizing donkey milk throughout lactation. To our knowledge, this is the first investigation to combine a large sample size with the simultaneous quantification of detailed chemical composition and key immunological proteins across multiple lactation stages. By sampling milk from numerous jennies, we intentionally captured biological variability, enabling a more robust analysis of relationships between compositional and functional traits. Furthermore, the multivariate statistical approach revealed underlying connections and clustering patterns linking chemical constituents with bioactive and immunological parameters, providing a dynamic overview of the nutraceutical profile of donkey milk as lactation progresses.

Based on this framework, we hypothesized that both the chemical composition and concentrations of key bioactive proteins in donkey milk vary in a stage-dependent manner across lactation.

To test this hypothesis, the objectives of this large-scale study were to characterize the evolution of total solids, protein, fat, lactose, and ash from early to late lactation; quantify lactoferrin, α-lactalbumin, β-lactoglobulin, and lysozyme at defined lactation stages; perform multivariate statistical analyses to identify how specific components influence or co-vary with others across stages; and compare the obtained values with published reference ranges to refine current knowledge on donkey milk as a potential nutraceutical and functional food.

## 2. Materials and Methods

### 2.1. Ethical Considerations

The study was conducted in compliance with the European Directive 2010/63/EU on the protection of animals used for scientific purposes, as well as Romanian national legislation governing the welfare of animals in research [25]. Milk collection was performed during routine milking, using non-invasive manual techniques that did not cause pain, stress, or harm to the animals. The study protocol was reviewed and approved by the Bioethics Committee of the University of Life Science “King Mihai I” from Timisoara, Romania, approval code 201/09.03.2023.

### 2.2. Management Practices and Milk Sampling

Milk was collected from five commercial donkey farms in western and central Romania. The study enrolled 153 lactating jennies of the Romanian local breed [26], all managed under similar extensive, pasture-based systems. All jennies were multiparous (aged between 3 and 16 years) and clinically healthy, as confirmed by routine veterinary examinations. Housing consisted of traditional farm conditions with access to open-air paddocks, natural ventilation, and shaded areas. Their diet consisted of natural pasture during the warm season and meadow hay supplemented with cereal grains during the cold season. Clean drinking water was available ad libitum. Parity ranged 2–7, and the mean body weight at enrolment was 300 ± 20 kg. To align biological variation with downstream nutritional and bioactivity analyses relevant to human health, animals were grouped by days in milk (DIM) at each visit as follows: 1–3 DIM considered very early lactation (*n* = 30), 31–90 DIM—early lactation (*n* = 45), 91–150 DIM—mid lactation (*n* = 43), and 151–210 DIM—late lactation (*n* = 35). This resulted in a total of 153 samples analysed. Days in milk (DIM) were calculated from the documented foaling date in farm records. It is important to note that animals contributing samples to one lactation stage were not resampled at later stages. Each stage, therefore, represents a distinct group of independent individuals.

Milk samples were collected manually during the morning milking, under farm conditions, following hygienic procedures. The first 3–5 streams were discarded, and a mid-stream sample was collected directly into sterile containers, pre-labelled with coded IDs (farm, animal, date, time, DIM). Approximately 100 mL of milk was collected per animal. Immediately after collection, samples were placed in isolated coolers with ice packs (≤4 °C) and transported to the Food Control Laboratory, Faculty of Veterinary Medicine, Timișoara, within two hours. Upon arrival, each sample was divided into two 50 mL aliquots: one aliquot was immediately processed for chemical analysis, while the other was stored at 2–4 °C for 3–4 days until all samples were collected for subsequent analysis using ELISA kits to quantify bioactive components. All analyses were performed on individual samples, without pooling, to preserve biological variation.

### 2.3. Determination of Milk Chemical Composition

The chemical composition of DM was determined for all samples, covering the defined lactation stages. The analyses targeted the main nutritional parameters: protein, fat, lactose, and total solids. Measurements were carried out using a Lactoscope FT-IR milk analyzer (Delta Instruments, Drachten, The Netherlands), which operates on mid-infrared spectroscopy in accordance with ISO 9622/IDF 141:2013 [27]. The equipment was calibrated daily with standardized milk solutions supplied by the manufacturer, and periodic performance checks were performed to ensure accuracy. Ash content was calculated by difference, subtracting the values of protein, fat, and lactose from total solids [28].

All determinations were performed in duplicate to minimize technical error. The mean of the two replicates was used for statistical analysis. If discrepancies greater than 5% between replicates occurred, the analysis was repeated until concordant results were obtained.

### 2.4. Quantification of Bioactive Proteins

Bioactive proteins were determined on the second aliquot. Due to its high biological activity and rapid degradation, colostrum was not included in the analysis, as it could not be reliably stored under refrigeration for the required period [29,30].

Concentrations of α-lactalbumin (ALA), β-lactoglobulin (BLG), lactoferrin (LF), and lysozyme (LZM) were measured using sandwich ELISA kits from MyBioSource (San Diego, CA, USA), validated for horse proteins: ALA (cat MBS105630), BLG (cat MBS067331), LF (cat MBS066136), LZM (cat MBS072858). Each kit included a precoated microelisa strip plate with specific capture antibodies, calibration standards, sample diluent, enzyme conjugates, wash solution, substrates, and stop solutions.

Assays were performed according to the manufacturer’s instructions. Milk samples were prepared and diluted appropriately to fall within the linear range of the calibration curve of each ELISA kit. All measurements were performed in duplicate, and inter-assay variation remained within the manufacturer-recommended limits. Standards and samples were pipetted into wells in duplicate (100 µL/well) on 96-well plates, incubated with capture antibodies, followed by washing to remove unbound proteins. Reactions were stopped with stop solution, and absorbance was read at 450 nm on a BioTek 800 TS microplate reader (Agilent BioTek Instruments, Winooski, VT, USA) within 5 min. Concentrations were calculated from the standard curves generated on each plate.

### 2.5. Statistical Analysis

The statistical analyses were conducted to assess the evolution of both chemical composition and bioactive compounds in donkey milk across different lactation stages. Descriptive statistics were first computed for each parameter, including mean, standard deviation (SD), minimum, maximum, and standard error of the mean (SEM). One-way ANOVA was applied to determine whether significant differences (*p*-value) existed across lactation stages. Post hoc analyses were performed using Tukey’s HSD test to identify which specific lactation stages showed significant pairwise differences. Pearson correlation coefficients were calculated to evaluate relationships among bioactive compounds (lactoferrin, alpha-lactalbumin, beta-lactoglobulin, and lysozyme) and between these and classical milk parameters (protein, fat, lactose, ash, and total solids). Multivariate techniques were also employed. PCA was conducted to explore the data structure and visualize patterns across lactation stages. K-means clustering and hierarchical clustering (dendrograms) were used to group variables and stages based on biochemical similarity. All statistical computations and visualizations were performed using Python (v3.11, Python Software Foundation, Wilmington, DE, USA; 2022).

## 3. Results

### 3.1. Chemical Composition Across Lactation Stages

**Figure 1:**
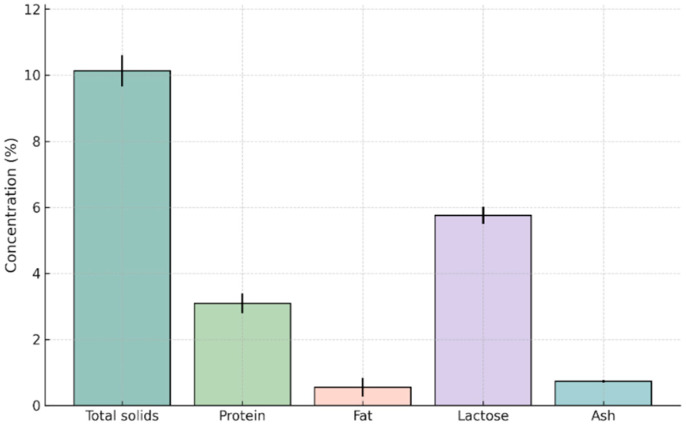
Chemical composition of donkey colostrum during very early lactation (1–3 days postpartum). Bars represent mean concentrations (%) of total solids, protein, fat, lactose, and ash (n = 30), with error bars indicating standard deviations. This profile illustrates the characteristic high-solid, high-protein, and low-fat composition of donkey colostrum at the onset of lactation.

During the very early lactation stage (1–3 days postpartum), donkey colostrum exhibited a composition characterized by elevated levels of solids and protein, moderate lactose, low fat, and stable mineral content (Figure 1, Table S1).

Total solids averaged 10.132 ± 0.473% (range: 9.47–11.63%), confirming that donkey colostrum is considerably richer in solids compared with mature stages. Protein content reached 3.091 ± 0.301% (range: 2.04–3.64%), accounting for a major share of total solids. Fat content was low overall (0.553 ± 0.288%, range: 0.25–1.58%), with relatively high variability among individuals. Lactose averaged 5.756 ± 0.256% (range: 5.08–6.23%), with limited variability. Ash content remained stable (0.732 ± 0.038%, range: 0.63–0.78%), reflecting a consistent mineral contribution. The stable mineral composition of donkey colostrum supports its function as a valuable supplementary source of nutrition, providing essential electrolytes and micronutrients that contribute to balanced dietary intake. Collectively, the 1–3 day postpartum stage is characterized by high solids and protein, modest lactose, and low fat. These findings suggest potential applications of donkey colostrum as a nutrient-dense and functionally active food for human nutrition and health.

**Figure 2:**
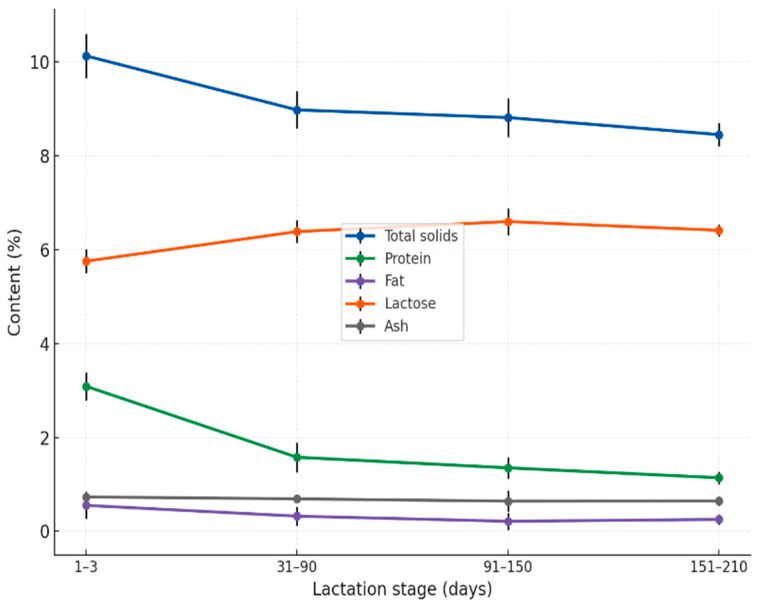
Chemical composition of donkey milk across lactation stages (1–3, 31–90, 91–150, and 151–210 days postpartum). Values represent mean concentrations (%) of total solids, protein, fat, lactose, and ash, with error bars indicating standard deviations (SDs). The trends illustrate the characteristic decline in total solids and protein, the consistently low fat content, the relative stability of ash, and the increase and stabilization of lactose as lactation progresses.

Mature donkey milk exhibited marked differences in nutrient composition across lactation stages, as shown in Figure 2. Total solids declined gradually during lactation, averaging 8.980 ± 0.396% at 31–90 days, 8.817 ± 0.416% at 91–150 days, and 8.454 ± 0.250% at 151–210 days (*p* < 0.001). Protein content followed a similar decreasing trend, with mean values of 1.578 ± 0.314%, 1.353 ± 0.229%, and 1.141 ± 0.131% for the respective stages (*p* < 0.001). Fat levels remained consistently low, but showed significant variation across lactation (*p* < 0.05), decreasing from 0.323 ± 0.205% at 31–90 days to 0.214 ± 0.188% at 91–150 days, and slightly increasing to 0.253 ± 0.110% in late lactation (151–210 days). Lactose exhibited the opposite pattern, increasing to 6.387 ± 0.240% in early lactation, reaching its highest mean value at 6.600 ± 0.287% in mid-lactation, and stabilizing at 6.415 ± 0.128% during the late stage (*p* < 0.001). Ash content remained relatively stable throughout the entire period, with mean values of 0.692 ± 0.048%, 0.643 ± 0.224%, and 0.645 ± 0.095% across the three stages (*p* > 0.05), indicating a consistent mineral contribution. These compositional dynamics suggest that DM can offer tailored nutritional benefits for different human dietary needs, ranging from immune support in specialized diets to low-protein, low-fat formulations for pediatric and clinical applications.

**Figure 3:**
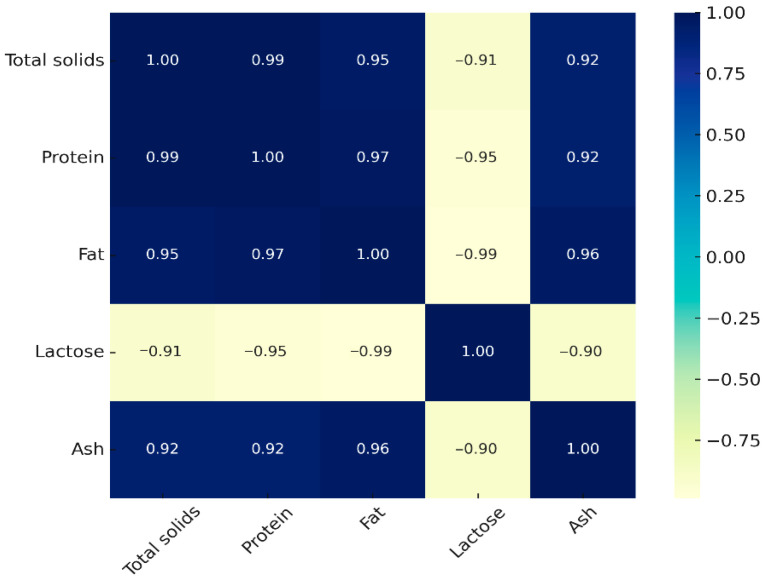
Heatmap of Pearson correlation coefficients among donkey milk components across lactation stages. Positive correlations are represented by blue tones and negative correlations by yellow tones, with numerical values indicating the strength of each association. The matrix highlights strong positive relationships among total solids, protein, fat, and ash, and strong negative relationships between lactose and the other major components.

Pearson correlation coefficients, calculated from mean values across lactation stages, revealed strong and nutritionally meaningful relationships (Figure 3).

Total solids were positively correlated with protein (r = 0.993), fat (r = 0.951), and ash (r = 0.922), while negatively associated with lactose (r = −0.914). This indicates that the nutrient density of donkey milk is primarily determined by its protein and fat fractions, whereas an increase in lactose corresponds to a physiological dilution of other macronutrients. Protein showed a strong association with fat (r = 0.974) and a positive correlation with ash (r = 0.921), but an inverse correlation with lactose (r = −0.954). From a nutritional perspective, this pattern underlines that the higher the protein content, the greater the contribution of essential minerals, while higher lactose concentrations occur in milk with reduced protein. Fat was strongly associated with ash (r = 0.958) and negatively correlated with lactose (r = −0.987). This suggests that, although donkey milk is naturally low in fat, its lipid fraction contributes proportionally to the mineral content, reinforcing the functional nutritional role of fat–mineral interactions. Ash showed consistent positive correlations with protein (r = 0.921), fat (r = 0.958), and total solids (r = 0.922), and a negative correlation with lactose (r = −0.904). This demonstrates that the mineral fraction of donkey milk follows the same compositional dynamics as solids, protein, and fat. Such consistency ensures a stable mineral contribution for human nutrition, even when lactose concentrations increase during later stages of lactation. The correlation analysis confirms that the decline in solids across lactation is mainly driven by reductions in protein and fat, whereas lactose rises inversely.

### 3.2. Bioactive Compounds Across Lactation Stages

**Figure 4:**
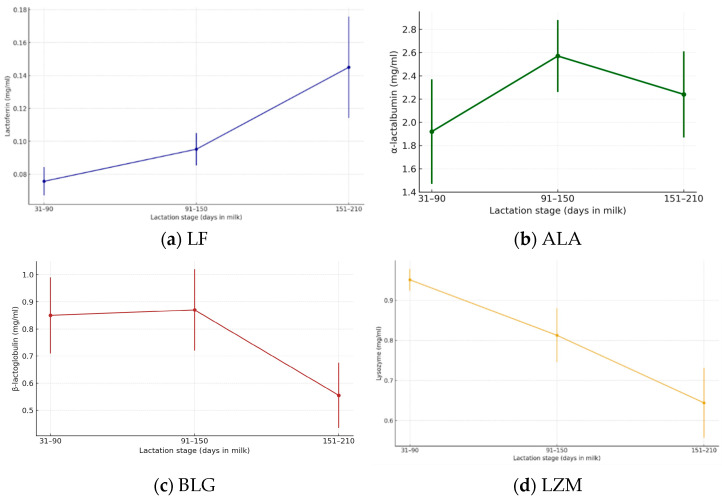
Concentrations of major bioactive proteins in donkey milk across lactation stages. Data are presented as mean ± standard deviation (SD): (**a**) Lactoferrin (LF) shows a progressive increase with advancing lactation. (**b**) α-Lactalbumin (ALA) rises toward mid-lactation and declines thereafter. (**c**) β-Lactoglobulin (BLG) remains relatively stable between early and mid-lactation before decreasing in late lactation. (**d**) Lysozyme (LZM) exhibits the highest levels in early lactation, followed by a gradual decline toward late lactation.

The concentrations of immune-active compounds (LF, ALA, BLG, and LZM) were evaluated across three lactation stages: 31–90 days, 91–150 days, and 151–210 days (Figure 4a–d).

Statistical analysis confirmed a significant overall effect of lactation stage on LF concentration (ANOVA, *p* < 0.001). Post hoc testing revealed significant differences between all stages: 91–150 days vs. 31–90 days (*p* < 0.05), 151–210 days vs. 31–90 days (*p* < 0.001), and 151–210 days vs. 91–150 days (*p* < 0.001). These findings highlight a progressive rise in lactoferrin concentration across lactation, with the most pronounced increase observed in the late stage. Such results are of particular importance for human nutrition and health, given the antimicrobial and immunomodulatory functions of this bioactive protein.

The global effect of lactation stage on ALA levels was statistically significant (ANOVA, *p* < 0.001). Post hoc analysis indicated that mid-lactation (91–150 days) had significantly higher concentrations compared with both early (31–90 days, *p* < 0.001) and late stages (151–210 days, *p* < 0.05). Moreover, late lactation levels were significantly lower than those of early lactation (*p* < 0.05). The results demonstrate a peak of α-lactalbumin secretion in mid-lactation, followed by a moderate decline in later stages. This dynamic pattern is relevant for human nutrition and health, given the established role of ALA in protein quality, digestibility, and bioactive peptide release.

The influence of lactation stage on BLG content was highly significant (ANOVA, *p* < 0.001). Post hoc comparisons revealed that late lactation (151–210 days) showed significantly lower concentrations than both early (31–90 days, *p* < 0.001) and mid-lactation (91–150 days, *p* < 0.001). In contrast, no meaningful difference was detected between early and mid-lactation stages (*p* > 0.05). These results indicate that β-lactoglobulin levels remain stable during the first half of lactation and decline significantly in the later phase. This trend is nutritionally relevant, as BLG is a key whey protein with allergenic potential in humans, and its lower levels towards the end of lactation may influence the tolerability and functional properties of donkey milk.

The statistical test confirmed a strong overall influence of lactation stage on LZM content (ANOVA, *p* < 0.001). Post hoc comparisons highlighted significant differences between all pairs: early vs. mid-lactation (*p* < 0.001), early vs. late (*p* < 0.001), and mid vs. late (*p* < 0.001). Lysozyme was most abundant during early lactation, with a progressive and statistically significant decline in the following stages. This trajectory is noteworthy for human nutrition and health, as this compound is a key antimicrobial protein that contributes to the protective and functional value of donkey milk.

All the data are summarized in Table 1.

### 3.3. Multivariate Statistical Analysis

A multivariate statistical approach was used to characterize donkey milk composition across three lactation stages (31–90, 91–150, and 151–210 days). By integrating univariate analyses with clustering, correlation, regression, and principal component analysis (PCA), we identified consistent stage-specific patterns and key interrelationships among bioactive proteins and major compositional traits.

The extended heatmap of absolute concentrations confirmed distinct trends across the lactation timeline. LZM and total protein showed their highest values in early milk, followed by a steady decline. In contrast, LF increased progressively, reaching maximum concentrations in late lactation. ALA peaked at mid-lactation, while BLG was also higher at this stage but decreased sharply by the end. Fat content reached its minimum in mid-lactation with a slight rebound later, whereas lactose remained relatively stable across all stages. Total solids mirrored the protein decline, highlighting a gradual dilution of the milk matrix in later stages.

**Figure 5:**
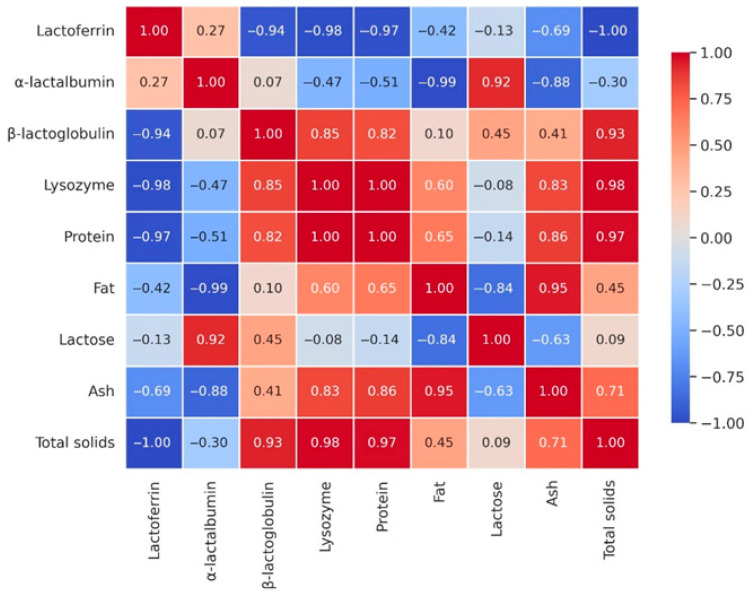
Pearson correlation heatmap of donkey milk parameters across lactation stages. Positive correlations are shown in blue and negative correlations in red, with color intensity reflecting the magnitude of each relationship. Numerical values indicate correlation coefficients, highlighting strong positive associations among protein, lysozyme, β-lactoglobulin, ash, and total solids, and strong negative associations between lactose and most other milk components.

Pearson’s correlation matrix (Figure 5) revealed strong co-variation between LZM and total protein (r = +0.998) as well as between lysozyme and total solids (r = +0.984). Conversely, LF was negatively correlated with total solids (r = −0.999), reflecting its opposite trend relative to most bulk protein fractions. Fat showed a marked negative correlation with ALA (r = −0.987). These associations highlight coordinated shifts in the bioactive and nutritional profile of milk during lactation.

**Figure 6:**
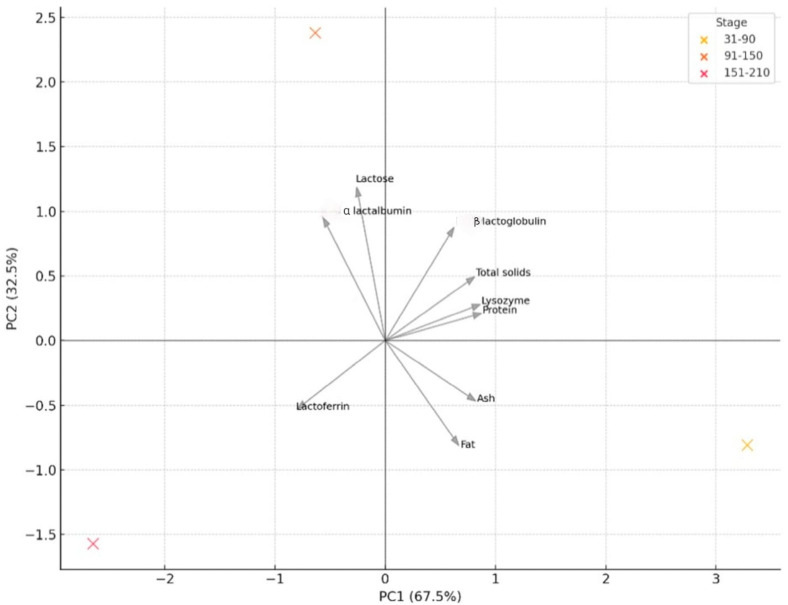
Principal component analysis (PCA) biplot of donkey milk composition across lactation stages. Points represent the distribution of samples from each lactation stage (31–90, 91–150, and 151–210 days in milk), while arrows indicate the contribution and direction of individual milk components to the principal components. PC1 and PC2 jointly explain the major sources of variability in the dataset, illustrating how compositional parameters cluster and shift across lactation.

The PCA biplot combines information about the distribution of lactation stages (as points) and the influence of each variable (as arrows) in the reduced two-dimensional space defined by the first two principal components (PC1 and PC2) (Figure 6). PC1 distinguished early milk (31–90 days), characterized by high protein, lysozyme, and solids, from late milk (151–210 days), which was enriched in LF but depleted in protein. PC2 captured the contribution of whey proteins (ALA, BLG) and fat, highlighting the distinct peak of α-lactalbumin in mid-lactation. The positioning of lactation stages along these axes emphasized a progressive compositional shift over time.

**Figure 7:**
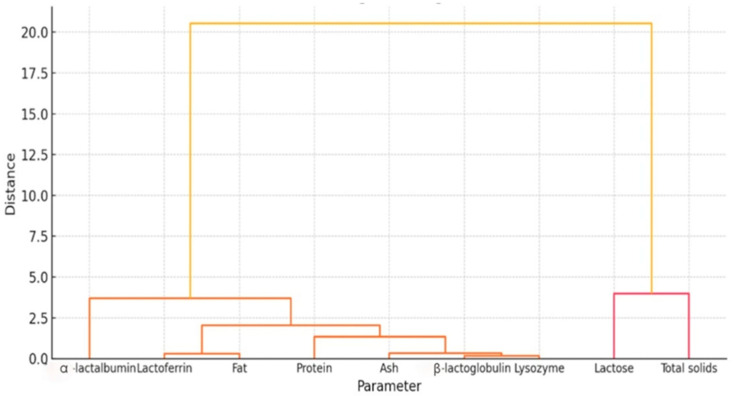
Hierarchical clustering dendrogram of donkey milk parameters. The analysis groups compositional and bioactive variables based on their similarity patterns, revealing two major clusters: one dominated by lactose and total solids, and another comprising protein, fat, ash, lysozyme, β-lactoglobulin, lactoferrin, and α-lactalbumin. The clustering structure reflects how milk components co-vary across lactation, highlighting distinct associations that differentiate early lactation profiles from those observed in mid and late stages.

Hierarchical clustering (Figure 7) supported previous findings from PCA and correlation analysis, offering another perspective on milk composition dynamics. Early lactation (31–90 DIM) formed a separate cluster, confirming its unique biochemical profile, while mid (91–150 DIM) and late (151–210 DIM) lactation grouped together due to greater similarity. On the variable axis, lysozyme, protein, and total solids clustered tightly, while α-lactalbumin grouped with β-lactoglobulin. Lactoferrin consistently emerged as the most distinct parameter, reflecting its inverse trajectory compared with other proteins. Fat and ash form a moderate cluster, somewhat independent from protein trends. Lactose appears loosely linked, reflecting its relatively stable concentration across stages. This hierarchical clustering analysis helps visualize how milk parameters group functionally and evolutionarily.

Regression models further confirmed stage-dependent changes. LZM (slope −0.153, R^2^ = 0.999, *p* = 0.0245) and total protein (slope −0.219, R^2^ = 1.000, *p* = 0.0109) both decreased linearly with lactation progression, whereas LF exhibited a strong opposing increase. These linear relationships suggest a systematic compositional reorganization of donkey milk across lactation; however, they are based on only three stage means and should therefore be interpreted in accordance.

These results demonstrate a progressive remodeling of donkey milk composition across lactation, reinforcing its nutritional significance. The variation in macronutrients and bioactive proteins across lactation stages supports its potential use as a stage-specific functional food in human health.

## 4. Discussion

The compositional profile of DM during early lactation and its subsequent evolution across stages highlights its role as a nutrient-dense product and a promising health-beneficial food for human consumption.

The compositional profile of donkey colostrum in the very early lactation stage highlights its properties as a high-value nutritional fluid enriched in solids, with moderate lactose, low fat, and stable mineral content, reflecting its vital contribution to neonatal immunity and nutrition. Our findings align closely with previous studies reporting elevated protein and mineral levels in early lactation [31,32,33]. Specifically, the protein content in the very early lactation days was more than double the levels typically found in later stages. Similarly, total solids and ash content were elevated, in agreement with the trends observed by Fantuz et al. [34] and Meena et al. [35], who highlighted the enriched mineral content of donkey colostrum. In contrast, lactose levels remained relatively low, which is a recognized feature of equid colostrum, as carbohydrate synthesis and water content increase progressively after the initial days [36,37]. The lipid fraction is consistently low (~0.16–1.16%), distinguishing donkey colostrum from ruminant milks where fat is a major energy source [31,38].

Significant variations in DM composition across lactation stages confirm that it is an adaptive secretion with changing nutritional and biofunctional properties. Our results align with studies reporting that total solids, protein, and fat decrease progressively after colostrum, while lactose increases and then stabilizes, and ash remains relatively constant [19,33,39].

Total solids decreased from 10.13% in early lactation to 8.45% in late lactation, a relative drop of approximately 16.6%. This decline aligns with values reported by Guo et al. [31], who found an average of 9.53% in Chinese donkeys, and with Malacarne et al. [32], who reported decreasing solids across lactation in Ragusano donkeys. Similar observations have been made in Cypriot and Balkan breeds [37,38], reflecting the universal trend of milk dilution as lactation progresses. Protein followed a parallel trajectory, decreasing from 3.09% to 1.14%, which mirrors reductions documented in multiple studies [33,35,39]. The reduction is characteristic of advancing lactation, as the synthesis of nitrogenous compounds diminishes over time, a phenomenon widely observed in mammalian lactation [40]. Fat content exhibited a modest increase during mid-lactation (from 0.55% to 0.68%) before plateauing at 0.25% in late lactation. While absolute values remain low compared to bovine milk, these fluctuations are within the expected range [41]. Some studies, such as those by Martini et al. [36] and Cunsolo et al. [42], suggest that dietary factors and metabolic state influence fat secretion, potentially accounting for variability. Others, including Aspri et al. [38], report relative stability, indicating breed or management-specific effects. Lactose content shows relative constancy across lactation, varying from 5.76% at the 1–3 day lactation stage to 6.42% in late lactation. This stability has been widely documented [1,19,31,39,43] and is attributed to the essential osmotic role of lactose in maintaining milk volume. Ash values remain stable across lactation, typically ~0.40% [43,44], ensuring a consistent supply of electrolytes and micronutrients. Notably, DM ash values are typically higher than those of human milk, making them an important nutritional attribute when considering substitution or fortification purposes [4].

Correlation analysis across lactation stages revealed clear interrelationships among DM components that define its evolving nutritional profile. Total solids were strongly correlated with protein and fat, whereas lactose showed an inverse association, reflecting the physiological transition from protein-rich early milk to lactose-dominant mature milk. Protein was also positively associated with ash, indicating that higher protein levels coincide with greater mineral contributions, consistent with previous reports by Malacarne et al. [32] and Živkov Baloš et al. [37]. This coupled enrichment highlights the nutritional relevance of colostrum as a source of immune-active proteins and essential micronutrients. Protein and lactose maintained a robust inverse relationship, in line with earlier findings [31,45]. This compositional shift has clear nutritional significance: in early lactation, the higher protein content contributes to immune protection and antimicrobial defence, whereas in later stages, increased lactose and reduced protein content enhance digestibility and favours the growth of beneficial gut microbiota [46]. Although DM is generally low in fat, its positive correlation with ash suggests that lipids may contribute to mineral stability, as also observed by Li et al. [47]. This property is particularly relevant for individuals requiring reduced caloric intake, as DM continues to provide consistent mineral contributions. Overall, these interdependent compositional shifts emphasize that donkey milk is not a uniform food but a dynamic product whose evolving profile across lactation can be strategically harnessed to support human health [48,49].

While the chemical composition defines the nutritional basis, the profile and variation of its bioactive proteins reveal its functional and health-promoting potential.

Donkey milk is naturally rich in bioactive compounds such as lactoferrin, α-lactalbumin, β-lactoglobulin, and lysozyme, which contribute to its functional and health-promoting properties. Lactoferrin is a multifunctional iron-binding glycoprotein found in milk that exerts strong antimicrobial, antiviral, anti-inflammatory, immunomodulatory, and anticancer properties. In humans, LF contributes to gut barrier integrity, supports mucosal immunity, and has been studied for roles in metabolic regulation, bone health, and cancer prevention [50,51,52]. ALA is a whey protein that, in the mammary gland, functions as the regulatory subunit of lactose synthase, thereby controlling lactose production and indirectly influencing milk volume and osmotic balance. Beyond this, it is rich in essential amino acids (notably tryptophan) and can yield bioactive peptides with antimicrobial, anti-stress, and antitumor effects. In human nutrition, ALA has been associated with improved amino acid balance, enhanced gut tolerance, and potential benefits for mood and cognitive function via tryptophan availability [52,53,54]. BLG is the major whey protein in many non-human milks, with the capacity to bind small hydrophobic molecules such as fatty acids and vitamins, suggesting a role in transport or stabilization of micronutrients. In a human health context, its binding properties and interactions with the gut and immune system have been explored to understand nutrient delivery and immunogenic risk [14,55]. Lysozyme is an antimicrobial enzyme that cleaves the β (1 → 4) glycosidic bonds in bacterial peptidoglycan, particularly targeting Gram-positive bacteria. In milk, LZM contributes to the innate antimicrobial defence of the neonate’s gut by reducing pathogenic bacterial load and shaping the microbiota. In human health, this compound supports mucosal immunity, contributes to control of infections, and may exert anti-inflammatory effects in the gastrointestinal tract [14,56,57].

Our results demonstrated distinct patterns: LF concentrations increased significantly across lactation, ALA peaked at mid-lactation, BLG remained stable until mid-lactation and then declined in late lactation, while LZM was highest in early lactation and decreased progressively. These findings are in line with the general patterns reported for donkey milk whey proteins in the literature, while also providing additional detail on how their concentrations shift across lactation [18]. LF showed a clear stepwise rise as lactation advanced, which agrees with reports by Vincenzetti et al. [18] and Gubić et al. [58], who described values ranging from 0.08 to 0.25 g/L in donkey milk, with indications of increased abundance later in lactation. In contrast, Guo et al. [31] reported relatively stable LF concentrations around 0.08 g/L, which may reflect breed or methodological differences. The rise observed in our study suggests an adaptive role of LF in maintaining antimicrobial defence when concentrations of other protective proteins, such as LZM, are declining. ALA displayed a mid-lactation peak, consistent with observations by Vincenzetti et al. [57] and Polidori and Vincenzetti [29], who reported values of 1.8–2.0 g/L in mature donkey milk with some fluctuations across stages. The higher levels at mid-lactation may be linked to increased lactose synthesis and metabolic activity of the mammary gland during the period of maximum milk yield. The subsequent decline toward late lactation resembles patterns described by D’Alessandro et al. [19], who observed stage-related variability but noted that ALA is generally well maintained compared to other fractions. BLG remained unchanged between early and mid-lactation but decreased significantly after 150 days. This pattern is supported by Gubić et al. [58], who documented values between 0.26 and 0.40 g/L with a declining trend over time. Similarly, Vincenzetti et al. [18] highlighted that BLG, though present in donkey milk, is relatively low compared to bovine milk and diminishes in later stages. The reduction observed in our study further supports the view that donkey milk becomes less allergenic as lactation progresses, since BLG is absent in human milk and considered a major bovine allergen [59,60]. LZM, by contrast, was highest in early lactation and declined significantly in mid and late lactation, matching the reports of Martini et al. [15], who measured values between 1.0 and 4.0 g/L with a progressive decrease over time. Jasmine et al. [61] also confirmed that DM contains exceptionally high lysozyme, up to 30–50 times higher than human milk, but that levels fall with advancing lactation.

Together, these patterns indicate a dynamic adjustment of the bioactive protein profile throughout lactation. The reciprocal trends of LF and LZM suggest functional compensation to ensure sustained antimicrobial protection, while ALA and BLG fluctuations reflect changes in metabolic demands and allergenic potential. Our results, therefore, complement and extend previous findings by demonstrating coordinated shifts in all four major bioactive proteins over an extended lactation window.

The compositional dynamics of donkey milk across lactation are not only stage-specific but also functionally significant, revealing interconnected shifts in bioactive and nutritional compounds that may support differentiated applications in human health. Multivariate analysis, supported by heatmaps, correlation matrices, and PCA, clarified how specific compounds co-vary in time and structure the biochemical identity of milk at each stage.

In early lactation (31–90 days), DM displays the highest levels of total protein, LZM, and total solids, forming a tightly intercorrelated cluster. The exceptionally strong positive correlations between lysozyme and both protein and solids suggest that its abundance is directly linked to the total proteome of early milk. In fact, LZM activity in donkey milk has been reported to peak in earlier lactation stages, supporting its role in antimicrobial defence [62]. This early-stage profile is biologically optimized to support mucosal immunity and microbial defence in the neonatal gut, and its integration into human health strategies could benefit individuals in need of enhanced immunoprotection. Immunocompromized patients, those recovering from gastrointestinal infections, or children with recurrent respiratory or enteric illnesses may particularly benefit from the antimicrobial and anti-inflammatory synergy of this protein-rich, lysozyme-dense milk matrix. The co-expression of total protein and solids also indicates high nutrient density, potentially useful in cachectic states or nutritional rehabilitation. Given the mild fat content at this stage, formulations may require lipid enrichment for complete energy balance, but the bioactivity profile remains unmatched across the lactation window.

The mid-lactation phase (91–150 days) marks a turning point in milk composition. ALA and BLG both peak, correlating with moderate fat levels and a relatively stable lactose content. Notably, fat exhibits a strong inverse correlation with ALA, suggesting an energetic trade-off in milk composition. This compound has been associated with improved sleep, cognitive performance, and emotional regulation in humans; dietary α-lactalbumin enhances the ratio of tryptophan to other large neutral amino acids, supporting central serotonin synthesis [52]. In animal studies, ALA enrichment increased systemic tryptophan and brain serotonin levels, which may underpin cognitive and mood benefits [53]. BLG also serves as a carrier of hydrophobic nutrients such as retinol and fatty acids, facilitating their bioavailability [18]. Thus, mid-lactation milk may offer a balanced nutritional profile appropriate for pediatric formulas, patients with mood or sleep disorders, or individuals needing precise protein-energy modulation—such as in metabolic syndrome, hepatic compromise, or recovery from major surgery.

In late lactation (151–210 days), the dominant shift is the progressive increase in LF, which emerges as the most compositionally distinct compound. Correlation analysis reveals a strong negative association between LF and both total solids and protein, underlining a biochemical transition in which immune–regulatory functions remain active despite a declining bulk nutrient load. Lactoferrin plays key roles in iron metabolism, host defense, and inflammatory modulation, and has been implicated in therapies for anemia, gut inflammation, and oncological support [49,50]. DM is recognized as “richer in lactoferrin and lysozyme” relative to cow milk, reinforcing its potential antimicrobial and immunoprotective functions [6]. The milk at this stage, while lower in total protein, may therefore be ideally suited for adults managing low-grade inflammation, iron overload, or requiring immune modulation with minimal nitrogen burden—such as patients with chronic kidney disease or low-grade colitis. The stable ash and lactose content in this stage ensures continued electrolyte support and energy provision, further supporting its potential as a maintenance-stage clinical food.

What emerges from these multivariate associations is not only a dynamic map of milk composition, but a rationale for stage-specific human application. Early-stage milk is immunologically rich and nutrient-dense; mid-stage milk provides functional proteins with cognitive and metabolic relevance; late-stage milk emphasizes anti-inflammatory and iron-regulatory components with minimal protein overload. These differences are not merely additive but interactive—defined by the co-variation of compounds, as evidenced by strong correlations such as LZM–protein in early lactation, ALA–fat in mid-lactation, and LF–protein inverse in late lactation. Understanding these correlation patterns enhances the ability to design precision nutritional interventions, leveraging the natural compositional architecture of donkey milk.

A major strength of this study lies in its integrated evaluation of both chemical composition and bioactive proteins in donkey milk across multiple stages of lactation, providing a comprehensive dataset that bridges nutritional and functional aspects. Unlike many previous studies limited to a single lactation period or a single source of animals, our work monitored clinically healthy, multiparous jennies from different farms, ensuring both internal validity and broader applicability. Another important strength is the longitudinal approach, which captured dynamic changes from colostrum through late lactation in both macronutrients and protective proteins.

However, certain limitations should be acknowledged. The analysis was restricted to four major bioactive proteins—LF, ALA, BLG, LZM—while other relevant compounds such as immunoglobulins, lactoperoxidase, or bioactive peptides were not included. In addition, while the use of multiple farms increased representativeness, diet and environmental variation among farms may have introduced uncontrolled influences on milk composition. Nevertheless, we consider this variability as a strength rather than a limitation, as such inter-farm variation reflects real-world conditions, where consumers typically obtain donkey milk from diverse farms and geographical regions.

Future studies should include a wider range of bioactive compounds in DM, such as immunoglobulins, growth factors, and peptides, to build a more complete picture of its functional properties. Research should also extend to different donkey breeds and management systems to confirm whether the trends observed here apply under diverse conditions. Another important area is the development of processing and preservation methods that maintain the activity of bioactive proteins while ensuring milk safety for consumers. Finally, combining compositional analysis with functional tests and clinical trials will be essential to fully demonstrate the role of donkey milk as a nutraceutical and functional food with potential benefits for human health. Future research should integrate functional validation (e.g., immunomodulation assays, glycemic impact, microbiota shaping) with compositional staging to guide evidence-based inclusion of donkey milk into clinical diets. Such stratification may also inform industrial harvesting and processing protocols to preserve stage-specific bioactivity for maximal human benefit.

## 5. Conclusions

By linking chemical composition and bioactive protein dynamics, this study provides new quantitative evidence for the functional value of donkey milk. The results show that although macronutrients are progressively diluted with advancing lactation, bioactive proteins remain tightly regulated and contribute to its health-promoting potential. Specifically, lysozyme and total protein are most abundant in early lactation, α-lactalbumin and β-lactoglobulin peak during mid-lactation, and lactoferrin increases steadily toward late lactation, reflecting distinct physiological roles throughout the lactation cycle. These insights strengthen the scientific basis for considering donkey milk as both a nutraceutical and a functional food, and they open pathways for future clinical and nutritional applications in human health.

## Figures and Tables

**Table 1:** Bioactive compounds in donkey milk by lactation stage.

Parameter	Stage 31–90		Stage 91–150		Stage 151–210		*p* Value
	Mean ± SD	Min–Max	Mean ± SD	Min–Max	Mean ± SD	Min–Max	
Lactoferrin	**0.0757 ±** 0.0085	0.0598–0.0918	**0.0951 ±** 0.0098	0.0797–0.1087	**0.1449 ±** 0.0308	0.0801–0.1986	<0.001
α-lactalbumin	**1.9169 ±** 0.4541	1.1495–2.6298	**2.5856 ±** 0.3286	1.9768–3.1294	**2.2512 ±** 0.3091	1.8203–2.8015	<0.001
β-lactoglobulin	**0.8441 ±** 0.1402	0.5510–0.9945	**0.8671 ±** 0.1634	0.6110–1.1785	**0.5554 ±** 0.0988	0.4145–0.7460	<0.001
Lysozyme	**0.9513 ±** 0.0270	0.9087–0.9881	**0.8127 ±** 0.0673	0.7016–0.9098	**0.6440 ±** 0.0869	0.5204–0.8272	<0.001

## Data Availability

The original contributions presented in this study are included in the article and the Supplementary Material; further inquiries can be directed to the corresponding author.

## Supplementary Materials

The following supporting information can be downloaded at: https://www.mdpi.com/article/10.3390/foods14244284/s1, Table S1: Chemical composition of donkey milk across lactation stages.
