# Supplementary material for: Bioactive Protein Profile and Compositional Evolution of Donkey Milk Across Lactation Reflecting Its Nutritional and Functional Food Value
Source: Foods. 2025 Dec 12;14(24):4284. doi: 10.3390/foods14244284 (PMC12732461; doi:10.3390/foods14244284)
Supplement: Supplementary file 1 [file foods-14-04284-s001.zip › foods-4007529-supplementary.pdf]

Table S1.

Chemical composition of donkey milk across lactation stages

	Lactation Stage (days)				
Parameters (%) Mean±SD	**1–3**	**31–90**	**91–150**	**151–210**	***p*-value**
Total solids	10.132 ± 0.473	8.98 ± 0.396	8.817 ± 0.416	8.454 ± 0.25	0.0000
Protein	3.091 ± 0.301	1.578 ± 0.314	1.353 ± 0.229	1.141 ± 0.131	0.0000
Fat	0.553 ± 0.288	0.323 ± 0.205	0.214 ± 0.188	0.253 ± 0.11	0.0075
Lactose	5.756 ± 0.256	6.387 ± 0.24	6.6 ± 0.287	6.415 ± 0.128	0.0000
Ash	0.732 ± 0.038	0.692 ± 0.048	0.643 ± 0.224	0.645 ± 0.095	0.2642
